# Long-term Outcomes After Emergency Laparotomy: a Retrospective Study

**DOI:** 10.1007/s11605-022-05372-3

**Published:** 2022-06-13

**Authors:** Aura T. Ylimartimo, Sanna Lahtinen, Juho Nurkkala, Marjo Koskela, Timo Kaakinen, Merja Vakkala, Siiri Hietanen, Janne Liisanantti

**Affiliations:** 1 Medical Research Center of Oulu, Research Group of Surgery, Anesthesiology and Intensive Care Medicine, Oulu, Finland; 2 Department of Surgery, Oulu University Hospital, P.O.BOX 21, 90029 OYS Oulu, Finland; 3 Department of Anesthesiology, Oulu University Hospital, Oulu, Finland

**Keywords:** Emergency laparotomy, Long-term outcomes, Mortality

## Abstract

**Background:**

Emergency laparotomy (EL) is a common surgical operation with poor outcomes. Patients undergoing EL are often frail and have chronic comorbidities, but studies focused on the long-term outcomes after EL are lacking. The aim of the present study was to examine the long-term mortality after EL.

**Methods:**

We conducted a retrospective single-center cohort study of 674 adults undergoing midline EL between May 2015 and December 2017. The follow-up lasted until September 2020. The primary outcome was 2-year mortality after surgery. The secondary outcome was factors associated with mortality during follow-up.

**Results:**

A total of 554 (82%) patients survived > 90 days after EL and were included in the analysis. Of these patients, 120 (18%) died during the follow-up. The survivors were younger than the non-survivors (median [IQR] 64 [49–74] vs. 71 [63–80] years, *p* < 0.001). In a Cox regression model, death during follow-up was associated with longer duration of operation (OR 2.21 [95% CI 1.27–3.83]), higher ASA classification (OR 2.37 [1.15–4.88]), higher CCI score (OR 4.74 [3.15–7.14]), and postoperative medical complications (OR 1.61 [1.05–2.47]).

**Conclusions:**

Patient-related factors, such as higher ASA classification and CCI score, were the most remarkable factors associated with poor long-term outcome and mortality after EL.

## Introduction

Emergency laparotomy (EL) is a common operation with a high incidence of postoperative complications.^1–3^ Patients undergoing EL are often old and have significant comorbidities, exposing them to complications that lead to poor outcomes.^3–5^ Longer life expectancy and an aging population have resulted in an increasing need for emergency surgery within this patient group.^4,6^ Emergency situations do not allow for proper patient selection, but high-risk patients with low probability of survival should be noted. For example, the CELIOtomy score was developed to predict early postoperative mortality after EL in order to prevent futile surgical interventions.^7^

EL includes a wide range of different procedures, from minor to major, with high variation in surgical pathology. One-year mortality after EL has been reported to be 22–34%.^2,8–12^ Several studies have reported 30- and 90-day mortality after EL of 11–24%.^2,3,5,8,9,11–14^ However, outcome studies with a follow-up longer than 1 year after EL are limited.^8,15^ Long-term outcome has been reported to be associated with patient-related factors, such as comorbidities and medical complications, rather than operation-related adverse events.^16,17^ Therefore, our aim in the present study was to examine the long-term outcomes and the pre- and peri-operative factors associated with mortality after EL.

## Material and Methods

This observational, retrospective, single-center study was carried out at Oulu University Hospital, Finland. The study design was approved by the hospital administration (reference number 66/2018). According to local policy, no statement from the local ethics committee was required for a retrospective study.

### Patients

All patients (*N* = 674) who underwent midline EL between May 2015 and December 2017 were identified and reviewed from hospital discharge records. We included all eligible patients who survived > 90 days after EL, leaving a total of 554 patients in the final analysis. The exclusion criteria were age < 18 years, urgent or emergency cholecystectomy or appendectomy, or emergency or urgent laparotomy due to gynecological causes.

### Data Extraction

We collected the following data regarding patient demographics and peri-operative and postoperative variables: age, gender, diagnosis, type and duration of operation, antibiotic therapy, complication type, ICU and hospital length of stay (LOS), Charlson Comorbidity Index (CCI), American Society of Anesthesiologists (ASA) classification, and albumin, leukocyte, platelet, hemoglobin, and C-reactive protein (CRP) levels. Data were collected from electronic medical records, anesthesia charts, and operation charts. The operations were divided into three groups according to the urgency: emergency operation within 0.5–3 h after the surgical decision, very urgent operation within 3–8 h after the surgical decision, and urgent operation within 24 h after the surgical decision.

The complications were categorized according to a previously described protocol.^17^ The complications were classified as operation-related or medical complications. Operation-related complications included surgical site infection, fascial rupture, bleeding, seroma, anastamotic leakage, strangulation or herniation, and need for reoperation during the same hospital stay. Medical complications included pneumonia, respiratory dysfunction, pulmonary embolism, sepsis, acute kidney dysfunction, acute liver dysfunction, stroke, acute myocardial infarction, resuscitation, heart failure, and atrial fibrillation. Time of death was retrieved from the hospital’s medical records to assess the in-hospital, 30-day, 90-day, and 2-year mortality rates. The Population Register Center of Finland provided data concerning the dates of deaths of non-survivors. The survival follow-up lasted until September 23, 2020.

### Statistical Analysis

Statistical analyses were performed using SPSS for Windows (IBM SPSS Statistics for Windows, Version 27.0, Armonk, NY, USA). Categorical data are presented as numbers and percentages. Continuous variables are expressed as the medians with 25^th^ and 75^th^ percentiles [25^th^-75^th^]. Comparisons were performed using Pearson’s chi-squared for proportional data and the non-parametric Mann–Whitney *U* test for continuous data. The differences were considered significant at *p*-values < 0.05. Cox regression analysis was used to calculate odds ratios (ORs) and 95% confidence intervals (CIs) for death during the follow-up. All variables with univariate significance < 0.1 were entered into the model and retained in the model if the multivariate *p*-values were < 0.05 or if they had a significant impact on the log-likelihood function. Age, ASA class, smoking status, CCI, operation time, operation urgency category, operation diagnosis, pre-operative CRP, hemoglobin, albumin, and creatine were included in the Cox regression analysis. Kaplan–Meier survival curves were drawn for the clinically most interesting factors according to the Cox regression model. Due to the retrospective nature of the study, we did not conduct a statistical power analysis to assess sample size.

## Results

**Fig. 1:**
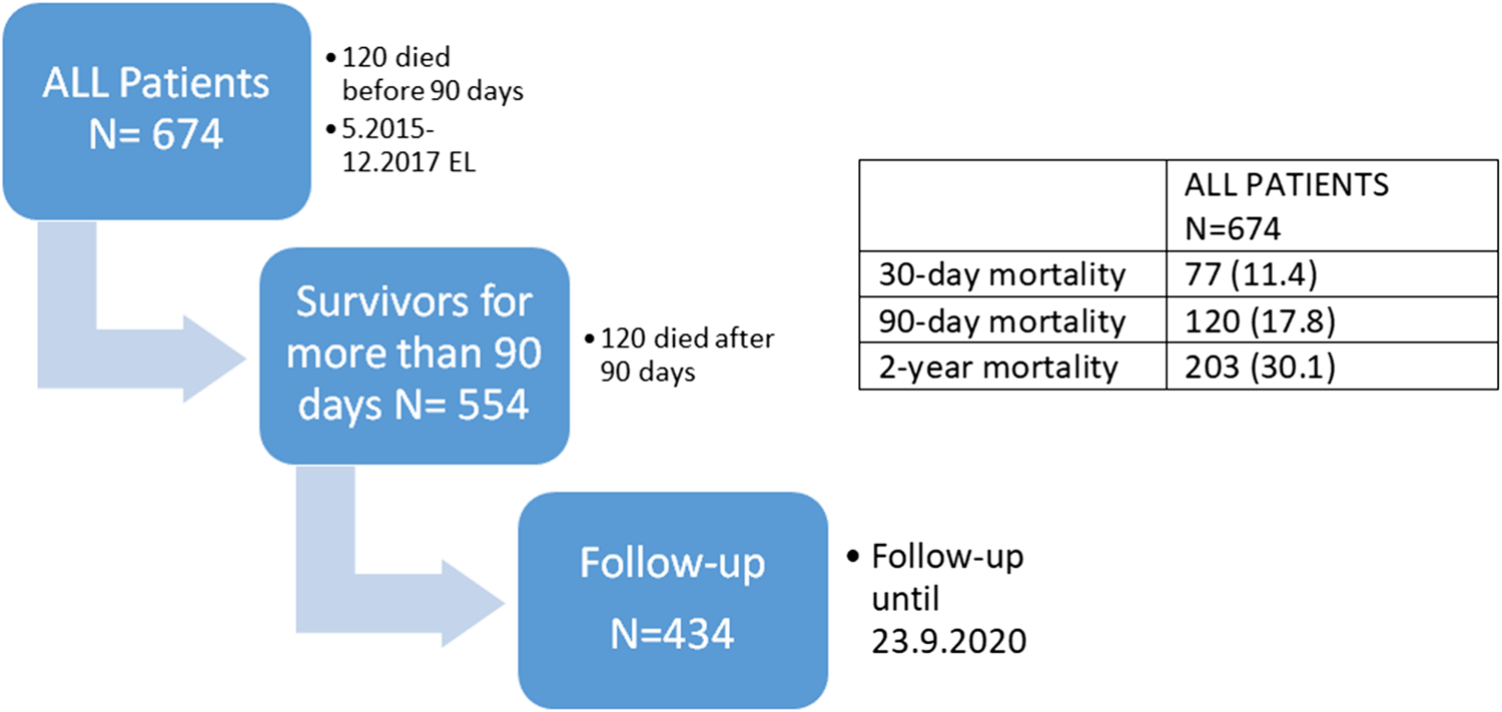
Study flow diagram

A total of 554 (82%) patients survived > 90 days after EL, including 120 (18%) who died during the follow-up (Fig. 1). The median follow-up for survivors was 3.1 [2.6–3.8] years. Non-survivors were more likely to be smokers (20.8% vs. 13.6, *p* = 0.050) and have higher median ASA classification (3 [3–4] vs. 3 [2–3], *p* < 0.001), higher median CCI scores (7 [4–8] vs. 3 [1–5], *p* < 0.001), history of malignancies (63.3% vs. 23.7%, *p* < 0.001), and advanced age (71 [63–80] years vs. 64 [49–64] years, *p* < 0.001) than the survivors (Table 1). Survivors were less likely to have hypertension (39.6% vs. 53.3%, *p* = 0.007), coronary artery disease (12.9% vs. 26.7% *p* < 0.001), kidney disease (2.1% vs. 10.0%, *p* < 0.001), chronic obstructive pulmonary disease (3.5% vs. 11.7%, *p* < 0.001), hypercholesterolemia (12.0% vs. 21.7%, *p* = 0.007), and dementia (2.3% vs. 7.5%, *p* = 0.006) than non-survivors.

**Table 1:** Characteristics of 554 patients who survived > 90 days after EL

Characteristic	Survivors *N* = 434	Non-survivors *N* = 120	*p*-value
Age	64 [49–64]	71 [63–80]	< 0.001
Male gender	228 (52.5)	71 (59.2)	0.197
ASA class	3 [2, 3]	3 [3, 4]	< 0.001
Pre-operative weight, kg	73 [63–85]	72 [64–82]	0.431
Pre-operative BMI, kg/m^2^	25.5 [22.5–29.1]	25.4 [22.2–28.3]	0.518
Smoker	59 (13.6)	25 (20.8)	0.050
Excessive alcohol consumption	41 (9.4)	16 (13.3)	0.215
Pre-operative abdominal imaging with CT Native CT without contrast agent CT with contrast agent	113 (26.0) 247 (56.9)	31 (25.8) 61 (50.8)	0.964 0.236
History of abdominal surgery Previous abdominal surgery Reoperation during the same hospital stay	166 (38.2) 40 (13.8)	47 (39.2) 19 (15.8)	0.855 0.038
Malignancy * Colorectal cancer*	103 (23.7) *58 (13.3)*	76 (63.3) *29 (24.1)*	< 0.001 *0.004*
CCI	3 [1–5]	7 [4–8]	< 0.001
No chronic comorbidities	107 (24.7)	3 (2.5)	< 0.001
Underlying diseases Hypertension Coronary artery disease Mechanical aortic valve/TAVI FA Heart failure Kidney disease Diabetes II Diabetes I COPD Asthma Hypercholesterolemia Gout Psychiatric diagnosis Crohn’s disease Ulcerative colitis Dementia	172 (39.6) 56 (12.9) 6 (1.4) 41 (9.4) 16 (3.7) 9 (2.1) 65 (15.0) 6 (1.4) 15 (3.5) 55 (12.7) 52 (12.0) 8 (1.8) 40 (9.2) 10 (2.3) 8 (1.8) 10 (2.3)	64 (53.3) 32 (26.7) 2 (1.7) 17 (14.2) 8 (6.7) 12 (10.0) 23 (19.2) 5 (4.2) 14 (11.7) 11 (9.2) 26 (21.7) 4 (3.3) 13 (10.8) 1 (0.8) 2 (1.7) 9 (7.5)	0.007 < 0.001 0.817 0.135 0.156 < 0.001 0.266 0.053 < 0.001 0.294 0.007 0.321 0.594 0.307 0.898 < 0.006

Values are given as *n* (%) or median [25^th^ to 75.^th^ percentile]. *ASA*, American Society of Anesthesiologists; *BMI*, body mass index; *CT*, computed tomography; *CCI*, Charlson Comorbidity Index; *TAVI*, transcatheter aortic valve implantation; *FA*, atrial fibrillation; *COPD*, chronic obstructive pulmonary disease

Non-survivors were more likely to undergo surgery within 8–24 h than survivors (33.3% vs. 22.6%, *p* = 0.016). The duration of EL was longer (105 [72–152] min vs. 93 [60–136] min, *p* = 0.023) and more often associated with malignancy (16.7% vs. 4.8%, *p* < 0.001) or colorectal operation (54.2% vs. 43.1%, *p* = 0.031) in the non-survivors compared to the survivors (Table 2). The non-survivors also had higher median pre-operative CRP levels (61 [22–285] mg/l vs. 39 [5–159] mg/l, *p* = 0.004) and lower median pre-operative (117 [101–131] g/l vs. 127 [110–143] g/l, *p* < 0.001) and postoperative hemoglobin levels (103 [97–112] g/l vs. 112 [99–124] g/l, *p* < 0.001) compared to survivors. The non-survivors had a higher rate of medical complications (61.7% vs. 44.2%, *p* < 0.001) and ICU admissions (39.2% vs. 23.0%, *p* < 0.001) compared to survivors. Palliative (11.8% vs. 0%, *p* < 0.001) and oncological care (13.4% vs. 6.0%, *p* = 0.007) were also more common among the non-survivors. No difference was found between the two groups in ICU LOS, but the hospital LOS was longer among the non-survivors (14 vs. 9 days, *p* < 0.001; Table 3).

**Table 2:** Perioperative data for 554 patients who survived > 90 days after EL

	Survivors *N* = 434	Non-survivors *N* = 120	*p*-value
Operation time, min	93 [60–136]	105 [72–152]	0.023
Urgency v= Emergency (operation within 0.5–3 h) Very urgent (operation within 8 h) Urgent (operation within 24 h)	213 (49.1) 123 (28.3) 98 (22.6)	47 (39.2) 33 (27.5) 40 (33.3)	0.054 0.856 0.016
Operation diagnosis			
Malignancy/tumor	21 (4.8)	20 (16.7)	< 0.001
GI ulcer	34 (7.8)	9 (7.5)	0.904
Hernia	35 (8.1)	9 (7.5)	0.840
Vascular cause	25 (5.8)	3 (2.5)	0.149
HBP	8 (1.8)	2 (1.7)	0.898
Diverticulitis/colitis	33 (7.6)	4 (3.3)	0.097
Peritonitis	8 (1.8)	6 (5.0)	0.051
Ileus/occlusion	129 (29.7)	32 (26.7)	0.514
Injury	19 (4.4)	0	0.020
Other rare causes	11 (2.5)	4 (3.3)	0.633
Postoperative complication	65 (15.0)	19 (15.8)	0.817
Operation type			
Abdominal wall, mesentery, peritoneum, and greater omentum	190 (43.8)	39 (32.5)	0.026
Upper GI tract	32 (7.4)	10 (8.3)	0.725
Small intestine and colorectal surgery	187 (43.1)	65 (54.2)	0.031
HBP	3 (0.7)	0	0.361
GI complication	22 (5.1)	6 (5.0)	0.976

Values are given as *n* (%) or median [25^th^ to 75.^th^ percentile]. *GI*, gastrointestinal; *HBP*, hepatopancreaticobiliary

**Table 3:** Outcomes of 554 patients who survived > 90 days after EL

	Survivors *N* = 434	Non-survivors *N* = 120	*p*-value
Pre-operative CRP, mg/l	39 [5–159]	61 [22–185]	0.004
POD1 CRP, mg/l	160 [91–269]	192 [135–270]	0.224
Pre-operative hemoglobin, g/l	127 [110–143]	117 [101–131]	< 0.001
POD1 hemoglobin, g/l	112 [99–124]	103 [97–112]	< 0.001
Pre-operative leukocytes, × 10^9^/l	9.8 [6.9–13.9]	9.6 [6.9–12.5]	0.391
POD1 leukocytes, × 10^9^/l	9.9 [7.2–13.8]	9.7 [7.4–13.9]	0.971
Pre-operative albumin, g/l	29 [24–34]	26 [23–31]	0.002
POD1 albumin, g/l	25 [21–28]	23 [19–25]	< 0.001
Pre-operative creatinine, µmol/l	69 [55–92]	79 [55–121]	0.053
POD1 creatinine, µmol/l	65 [54–90]	72 [54–112]	0.099
Complications	213 (49.1)	80 (66.7)	0.001
Operation-related complications	120 (27.6)	43 (35.8)	0.082
Medical complications	192 (44.2)	74 (61.7)	< 0.001
Hospital LOS, days	9 [6–16]	14 [8–24]	< 0.001
Presurgery LOS, days	1 [0–3]	1 [0–4]	0.008
Postsurgery LOS, days	7 [5–13]	10 [6–17]	< 0.001
ICU admission	100 (23.0)	47 (39.2)	< 0.001
ICU LOS, days	5 [3–11]	6 [4–14]	0.198
Antibiotic therapy < 3 days 3–5 days 5–14 days > 14 days	317 (73.0) 15 (3.5) 31 (7.1) 108 (24.9) 163 (37.6)	104 (86.7) 5 (4.2) 5 (4.2) 40 (33.3) 41 (45.0)	0.002 0.712 0.242 0.064 0.495
Limitation of treatment DNR Other treatment restrictions Withdrawal from treatments Palliative care Oncological care	6 (1.4) 3 (0.7) 0 0 26 (6.0)	5 (4.2) 4 (3.4) 1 (0.8) 14 (11.8) 16 (13.4)	0.053 0.022 0.057 < 0.001 0.007
Discharge location Home Health center ward Central hospital Residential/nursing home Prison hospital	260 (59.9) 142 (32.7) 28 (6.5) 3 (0.7) 1 (0.2)	33 (27.5) 72 (60.0) 12 (10.0) 1 (0.8) 0	< 0.001 < 0.001 0.184 0.871 0.597

Values are given as *n* (%) or median [25^th^ to 75.^th^ percentile]. *CRP*, C-reactive protein; *POD1*, postoperative day 1; *LOS*, length of stay; *ICU*, intensive care unit; *DNR*, do not resuscitate

**Fig. 2:**
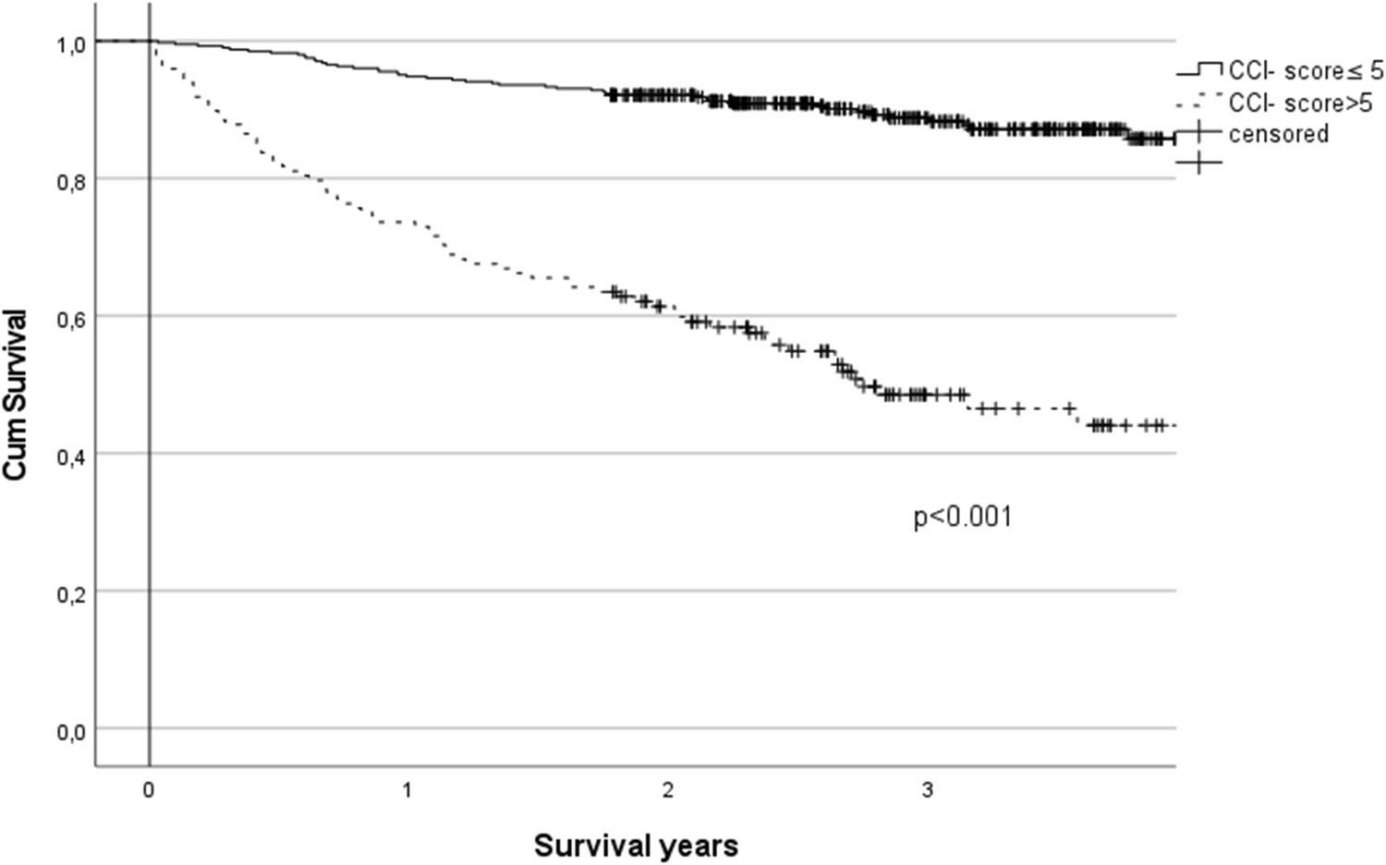
Kaplan–Meier survival curves for CCI scores in 554 patients who survived > 90 days after emergency laparotomy

**Fig. 3:**
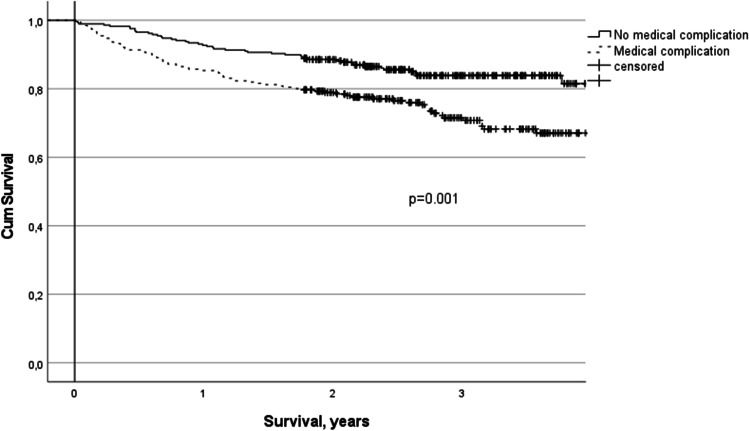
Kaplan–Meier survival curves for medical complications in 554 patients who survived > 90 days after emergency laparotomy

According to Cox regression analysis, ASA classification > 2 (OR 2.37, 95% CI 1.15–4.88, *p* = 0.019), CCI score > 5 (OR 4.74, 95% CI 3.15–7.14, *p* < 0.001), operation duration > 60 min (OR 2.21, 95% CI 1.27–3.83, *p* = 0.005), and recorded medical complications (OR 1.61, 95% CI 1.05–2.47, *p* = 0.030) were associated with mortality during the follow-up after EL (Table 4). A significant decline in survival was observed in patients with a CCI score > 5, and it lasted throughout the follow-up. When the patients with and without medical complications were compared, a decline in survival was observed at the beginning but plateaued after 2 years of follow-up (Figs. 2 and 3).

**Table 4:** Logistic Cox regression analysis of variables associated with mortality during follow-up in 554 patients who survived > 90 days after emergency laparotomy

	OR	95% CI	*p*-value
Duration of operation > 60 min	2.21	1.27–3.83	0.005
ASA > 2	2.37	1.15–4.88	0.019
CCI > 5	4.74	3.15–7.14	< 0.001
Medical complication	1.61	1.05–2.47	0.030
Operation-related complication	0.77	0.50–1.20	0.236
Age	1.00	0.98–1.01	0.641

*ASA*, American Society of Anesthesiologists; *CCI*, Charlson Comorbidity Index

## Discussion

The main finding of the present study is that the 2-year mortality after EL is nearly 20% in patients who survived at least 90 days after surgery. According to our results, poor outcome is mainly associated with patient-related factors, such as comorbidities. In particular, medical complications during the initial postoperative phase are associated with poor long-term outcomes.

This study is the first to demonstrate long-term survival after EL. Previous studies reported that mortality after EL is high (11–34%),^2,3,5,8–14^ but these reports focused on short-term survival. In the present study, most deaths occurred within 2 years after EL, which reflects the significance of the long-lasting recovery process; thus, studies reporting 90-day or 180-day mortality are too short-sighted to depict the long-term outcome. We demonstrated a mortality of 20% during the 2-year follow-up in the cohort of 90-day survivors. According to the multivariate analysis, mortality was strongly associated with CCI and ASA classifications, which indicates that, after the initial recovery, long-term survival is most likely dependent on comorbidities, not the acute disease. This finding is in line with previous studies, which indicated that higher ASA classification, low functional status, and presence of sepsis are associated with increased mortality.^3,8,18^

Other studies have reported that emergency general surgery is associated with high complication rates and death.^1,19,20^ The association between medical complications and poor outcomes is supported by previous findings in other patient groups.^16,17^ Havens et al. found that emergency general surgery patients are more prone to complications and likely to die within 30 days despite the patients’ pre-operative comorbidity and physiological status compared to elective patients.^21^ In our study, medical complications were a risk factor for mortality, even though we only included 90-day survivors. More than half of the non-survivors had a history of malignancy, which has been shown to be a risk factor for 30-day mortality after EL.^3,18,22^

Interestingly, the surgical procedure itself was not associated with mortality during follow-up, though a longer operation duration was associated with poor long-term outcome. The duration of the operation and its impact on postoperative morbidity has been examined extensively, but the association with mortality is less clear.^23^ A previous meta-analysis showed that the complication rate increases progressively with increasing operative time.^24^ Such complications include surgical site infection, wound infection or dehiscence, bleeding, pneumonia, urinary tract infection, and renal failure.^24^ However, the present study did not show an association between operation-related complications and mortality. Notably, we included only the 90-day survivors in the analysis, which may explain this result. Iversen et al. found that medical complications after EL for colon cancer were the major cause of death within 30 days of surgery, but operation-related complications did not increase mortality.^25^

Surgical management of acute abdominal pain aims for curative care and complete management of the underlying cause, but the complication rate and mortality rate are high. We showed that several patient-related factors are the predominant factors associated with poor outcome. Therefore, the pre-operative data should play a major role in decision-making, especially in frail patients and those of advanced age in order to avoid futile surgical interventions.^26^

Only a few prognostic scoring tools are available, such as the National Emergency Laparotomy Audit (NELA) and the CELIOtomy risk score calculators. The NELA requires recording several different values, making it impractical in a clinical environment. In addition, it is based on population statistics. However, these risk calculators may be useful in cases in which we have hours to discuss the options with patients and family. Reports from the NELA and Emergency Laparotomy Collaborative (ELC) show that using a care bundle may decrease mortality and hospital LOS after EL.^27,^^28^ The care bundle of the ELC includes six difference points: patient’s the national early warning score (NEWS)/the systemic response syndrome (SIRS) /arterial lactate level, detection of sepsis and early initiation of antibiotic therapy, admitting patient to the operating room within 6 h of the decision to operate, senior surgeon and anesthesiologist consult, monitoring cardiac output, and ICU for all EL patients.^27^ Standardization of treatment also improves prognosis in emergency surgery.^29^ In the future, combining different risk calculators and care bundles could improve the quality of care of EL patients.

The quality of life and life expectancy of a high-risk patient should be estimated before emergency surgery. The systematic review by Bouras et al. demonstrated that adverse surgical events have a negative effect on quality of life in general and gastrointestinal surgery.^30^ Complication rates are high after EL, and multicomorbidity and age increase the risk of complications. EL should not significantly impair the patients’ long-term quality of life. In some cases, withholding surgical treatment could be an option, but only one study has addressed this problem.^31^ We should recognize patients who do not benefit from surgery and offer them palliative care instead of curative treatment. However, there is no unambiguous answer to the question of which patients should not undergo surgery. Patient selection is challenging in emergency situations because it is not a real selection compared to patients undergoing elective surgery. One study has focused on quality of life after EL; Saunders et al. showed that patient-reported outcome measures from EL patients are a feasible measure, but recruitment bias occurred.^32^ Therefore, studies reporting this item are needed.

## Limitations

This study has several limitations. It is a single-center, retrospective, observational study, which limits the conclusions we can draw from the results. Due to the retrospective nature of the study, some patient data were missing, including data on the need for dialysis/continuous renal replacement therapy and parenteral nutrition. Moreover, we were not able to show any association between the examined factors and long-term mortality. Finally, we were not able to retrieve the causes of death, which would have increased the interpretability of the results. Further studies are needed to elucidate the proportion of deaths that were actually associated with the disease for which the patient underwent surgery or with the other comorbidities.

## Conclusions

The main finding of the present study is that the 2-year mortality among patients undergoing EL was nearly 20% for patients who survived at least 90 days after surgery. Postoperative medical complications, higher ASA class and higher CCI scores were associated with poor long-term outcomes after emergency laparotomy.
